# Illinois State Medical Society

**Published:** 1873-09

**Authors:** 


***Illinois State Medical Society.***

Secretary's Office,
No. 296 W. Monroe St.,
Chicago, July 1, 1873.

At the last Annual Meeting of the Illinois State Medical Society, held in the city of Bloomington, May 21st and 22nd, 1873, the following officers were elected for the ensuing year, and the following committees appointed to report at the next meeting of the Society, to be held in the city of Chicago, on the third Tuesday of May, 1874. T. D. Fitch, *Sec.*

Officers—President, T. F. Worrell, M.D., Bloomington ; 1st V. President, E. L. Holmes, M.D., Chicago ; 2nd V. Pres., S. H. Birney, M.D., Urbana ; Treasurer, J. H. Hollister, M.D., Chicago ; Permanent Sec., T. D. Fitch, M.D., Chicago ; Assistant Sec., Wm. E. Quine, M.D., Chicago.

STANDING COMMITTEES.

Board of Censors—David Prince, M.D., Jacksonville ; A. L. McArthur, M.D., Rockford ; J. L. White, M.D., Bloomington.

Committee of Arrangements—N. S Davis, M.D., Chicago ; Wm. E. Quine, M.D., Chicago ; Charles G. Smith, M.D., Chicago ; Edwin Powell, M.D., Chicago.

Committee on Practical Medicine—S. K. Crawford, M.D., Monmouth ; John Wright, M.D., Clinton, DeWitt county ; A. K. Van Horn, M.D., Jerseyville, Jersey county.

Committee on Surgery—W. P. Pierce, M.D., Lemont ; R. Roskotten, M.D., Peoria ; E. R. Willard, M.D., Wilmington.

Committee on Obstetrics—S. C. Plummer, M.D., Rock Island ; C. T. Horner, M.D., Naples ; Robert Boal, M.D., Peoria.

Committee on Drugs and Medicines—Wm. E. Quine, M.D., Chicago ; G. Wheeler Jones, M.D., Danville ; E. P. Cook, M.D., Mendota.

Committee on Necrology—G. W. Albin, M.D., Neoga ; J. O. Hamilton, M.D., Jerseyville ; J. K. Secord, M.D., Elmwood.

Committee on Ophthalmology—E. L. Holmes, M.D., Chicago ; J. P. Johnson, M.D., Peoria ; F. C. Hotz, M.D., Chicago.

Committee on Otology—Samuel J. Jones, M.D., Chicago ; Charles Hunt, M.D., Dixon ; Thos. Galt, M.D., Rock Island.

SPECIAL COMMITTEES.

Committee on Idiocy—C. T. Wilbur, M.D. Jacksonville.

Committee on Galvano-Therapeutics—David Prince, M.D., Jacksonville.

Committee on Local Functions of the Brain—Charles W. Earle, M.D., Chicago.

Committee on Phthisis Pulmonalis—J. M. Hutchinson, M.D., Chicago.

Committee on Stricture of Urethra—E. Andrews, M.D , Chicago.

Committee on Diseases of Respiratory Organs—Daniel Lichty, M.D., Rochelle.

DELEGATES TO THE AMERICAN MEDICAL ASSOCIATION.

Drs. A. H. Luce, Bloomington ; J. O. Hamilton, Jerseyville ; C. Goodbrake, Clinton ; E. P. Cook, Mendota ; W. P. Pierce, Lemont ; David Prince, Jacksonville ; C. T. Horner, Naples ; J. H. Hollister, Chicago ; W. W. McMann, Gardner ; S. H. Birney, Urbana ; Charles W. Earle, Chicago ; J. B. Walker, Mason City ; F. B. Haller, Vandalia ; W. E. Quine, Chicago ; E. R. Willard, Wilmington ; J. S. Whitmire, Metamora ; E. R. Travers, Amboy ; J. P. Mathews, Carlinville ; G. W. Hewitt, Franklin Grove ; D. W. Young, Aurora ; T. D. Fitch, Chicago ; O. W. Moore, Braceville ; John Lyttle, Leroy ; A. C. Rankin, Loda ; O. Everett, Dixon ; F. H. Davis, Chicago ; J. G. Curtis, Otterville; S. C. Plummer, Rock Island; F. M. Wilder, Chicago; J. L. White, Bloomington; A. L. McArthur, Rockford; N. B. Cole, Bloomington; R. E. McVey, Waverley; C. Armstrong, Gurley; Andrew McFarland, Jacksonville.

DELEGATES TO STATE MEDICAL SOCIETIES.

Indiana—Drs. George Wheeler Jones, and T. N. Booe, Loda.
Ohio—Drs. E. W Gray, Bloomington, and Ellsbury, Mason City.
Michigan—Drs. Moses Gunn, Chicago, and E. L. Holmes, Chicago.
Wisconsin—Drs. A. L. McArthur, Rockford, and Irwin, Loda.
Iowa—Drs. T. D. Fitch, Chicago, and Thos. Galt, Rock Island.
Missouri—Drs. C. R. Parke, Bloomington, and J. O. Hamilton, Jerseyville.
Minnesota—Drs. T. F. Worrell, Bloomington, and E. R. Willard, Wilmington.

